# Cross-Cultural Patterns in Dynamic Ratings of Positive and Negative Natural Emotional Behaviour

**DOI:** 10.1371/journal.pone.0014679

**Published:** 2011-02-18

**Authors:** Ian Sneddon, Gary McKeown, Margaret McRorie, Tijana Vukicevic

**Affiliations:** School of Psychology, Queen's University Belfast, Belfast, United Kingdom; Macquarie University, Australia

## Abstract

**Background:**

Studies of cross-cultural variations in the perception of emotion have typically compared rates of recognition of static posed stimulus photographs. That research has provided evidence for universality in the recognition of a range of emotions but also for some systematic cross-cultural variation in the interpretation of emotional expression. However, questions remain about how widely such findings can be generalised to real life emotional situations. The present study provides the first evidence that the previously reported interplay between universal and cultural influences extends to ratings of natural, dynamic emotional stimuli.

**Methodology/Principal Findings:**

Participants from Northern Ireland, Serbia, Guatemala and Peru used a computer based tool to continuously rate the strength of positive and negative emotion being displayed in twelve short video sequences by people from the United Kingdom engaged in emotional conversations. Generalized additive mixed models were developed to assess the differences in perception of emotion between countries and sexes. Our results indicate that the temporal pattern of ratings is similar across cultures for a range of emotions and social contexts. However, there are systematic differences in intensity ratings between the countries, with participants from Northern Ireland making the most extreme ratings in the majority of the clips.

**Conclusions/Significance:**

The results indicate that there is strong agreement across cultures in the valence and patterns of ratings of natural emotional situations but that participants from different cultures show systematic variation in the intensity with which they rate emotion. Results are discussed in terms of both ‘in-group advantage’ and ‘display rules’ approaches. This study indicates that examples of natural spontaneous emotional behaviour can be used to study cross-cultural variations in the perception of emotion.

## Introduction

One of the main questions facing those who study the expression and perception of emotion is the extent to which such processes are universal across cultures. There is no intuitively obvious answer to this question — humans share many broad characteristics and behaviours between cultures but equally, many more detailed aspects of our physical appearance, behaviour and cognition vary markedly across geography and time. Where do emotional signals fit into this pattern of similarity and variation? The history of research on this topic saw the popularity of the universalist and cultural relativist positions wax and wane throughout the twentieth century but, since the groundbreaking work by Ekman and colleagues [1], [2] and by Izard [3], a fairly coherent picture of the interplay between universal and cultural influences has emerged. This research has shown repeatedly that under a range of conditions, recognition of basic emotions through interpreting the facial expressions of members of different cultures reliably exceeds levels that would be expected by chance [4]. However, this work has also provided evidence that members of different cultures vary systematically in aspects of their interpretation of emotional expression [5]–[7]. The early explanation for such cultural differences was that, overlaid on the biologically based display and recognition system, a set of learned and culturally determined display rules operates to influence the frequency and intensity of emotional display. More recently, it has been suggested [8] that culture may also influence the frequency and intensity of emotional expression by affecting the way we understand the meaning of situations or by altering the frequency of occurrence of emotion inducing situations.

In an attempt to impose some structure on comparisons between cultures, four of Hofstede's [9] cultural dimensions have been used [10] as an explanatory framework to help understand the theoretical reasons for the emergence of display rules in certain cultures. Hofstede used a large scale values survey and identified the following salient dimensions: Power Distance (the degree to which a culture accepts large inequalities in power); Individualism (the degree to which a culture emphasises the relative importance of individual goals and independence over adherence to group norms and structures); Uncertainty Avoidance (the degree to which a culture feels threatened by ambiguity and has created beliefs and institutions to avoid them); and Masculinity (the degree to which a culture values traditional masculine values and makes clear differentiations between genders).

An initial series of hypotheses was proposed [6], [10] linking these dimensions with emotion display and observation rules. Cultures scoring high on Power Distance should exhibit lower levels of expression and perception of negative emotions. Cultures scoring high on Individualism should show higher levels of expression and perception of negative emotions. Cultures that show a high score on Uncertainty Avoidance should show lower levels of expression and perception of fear, and cultures that score high on Masculinity should show higher levels of gender difference in emotion expression and perception. Hofstede's dimensions were used [10] to re-analyze a mixture of emotion recognition scores and intensity ratings from four previous studies involving 15 cultures. Matsumoto's hypotheses for the emotion recognition scores can be summarized by the proposition that members of cultures in which an emotion is expressed less frequently (for whatever reason) will show correspondingly poorer recognition scores for facial expressions of that emotion – reflecting their relative lack of experience [11]. Subsequent studies, however, have offered little support for these predictions [12] although it has been suggested [11] that this may be at least partly due to measurement problems rather than a problem with the underlying theory. Matsumoto's hypotheses regarding the intensity ratings are clearly expressed in a study [13] of Japanese and American encoders and decoders. It is suggested that “... similar rules, much like Buck's (1984) [14] decoding rules, may exist concerning the perception of emotion. Display rules in Japan not only may attenuate their expressions of emotion, but may similarly downplay how emotional anyone else is seen to be. By this reasoning, the Japanese will perceive less intense emotion than Americans ...” (pp. 144–145). Although they offer some experimental support for this position [13], it seems a difficult hypothesis to explain in functional terms. If an individual from a culture that attenuates emotional expression experiences an intense emotion, this will typically be displayed at a lower intensity (obeying the display rule). If an individual from that culture observes a given level of emotional display, this will typically be perceived as reflecting a lower level of emotional experience (obeying the decoding rule). If we now combine the two rules it is clear that the observer ends up just being wrong about the emotional state of the displayer and is presumably ill equipped to make effective judgements about how to behave appropriately towards the displayer or about how the displayer is likely to behave towards them.

An alternative possibility [15] that seems easier to explain from a functional perspective, is that decoding rules might correct any distortion introduced by display rules, allowing a more accurate judgement to be made. However it has also been proposed [12] that decoding rules may affect only the reporting of perceptions and not the perceptions themselves — allowing individuals to respond in an appropriate manner to an emotional stimulus while reporting it as having a lower intensity. An alternative theoretical framework [12], [16] proposes that there is an ‘in-group’ advantage in recognition of emotions from facial expression and that accuracy in decoding the emotional expressions of people from other cultures will decrease with both geographical and cultural distance (as measured by discrepancies in Hofstede's dimension scores).

A resolution of the issues concerning display rules in cross-cultural perception of emotion has for a long time been prevented by the limited nature of the methodologies applied to the problem. The overwhelming majority of studies that comprise this research tradition have used what has been called the ‘standard method’ [17], which involves using posed still photographs of prototypical facial expressions. The few studies that have used spontaneous facial expressions indicate that recognition is typically lower than for posed photographs (for a summary see [18]). It has been argued [8] that this difference is easily explained by the diminished ‘signal clarity’ of spontaneous stimuli. Expressing emotions is only one aspect of facial behaviour, and in real situations, the presence of other facial behaviours may interfere with the clarity of the emotional signal. The careful pre-selection of the posed photographs used in most research has been criticised [17] as rendering them quite different from the facial expressions of emotion we encounter in everyday life. It is still not clear therefore to what extent the substantial body of results on the cross-cultural recognition of emotional expression can be generalised to real life situations, and researchers have been urged [18] to gather evidence from spontaneous dynamic stimuli.

**Figure 1:**
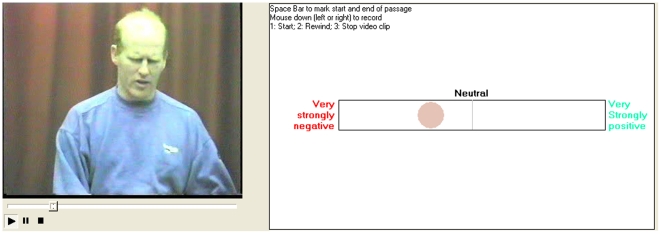
Feeltrace tool with a still image from a clip.

Recognising that many previous studies have sacrificed ecological validity for reliability, we have constructed a different type of study around a core of more natural dynamic stimuli. Use of such stimuli presents some challenges, however, in that we have no means of knowing objectively what emotional experience is being reflected by the facial expression displayed. Whilst it would conceivably be possible to obtain partial information via retrospective self report, that is not available in the present case as we have used stimuli from an existing database of emotional behaviour sequences — the Belfast Naturalistic Database. A further challenge is to capture the dynamic nature of observers' responses to the stimuli. Spontaneous dynamic stimuli are an extremely rich source of information with much it encoded in the dimension of time. To retain as much of the temporal response information as possible we used a variant of a computer based program called FeelTrace, Figure 1 [19]. The variant we employ allows participants to continuously record their rating of the strength of positive or negative emotion (valence) being displayed by the target individual in the stimulus clip. Thus, our focus is different from previous research on cross-cultural variation in perception of the facial expression of emotion. Rather than comparing rates of emotion recognition we are comparing temporal patterns of ratings of the strength of positive and negative emotions.

If the pattern of results from previous research, conducted on perception of static posed photographs of emotion, is to be reflected in the present study using natural dynamic stimuli, we would expect to see similarities in response patterns across cultures in combination with cultural variations in ratings of intensity. In a secondary hypothesis, if there is a link between display and decoding rules, as discussed above, we would expect that the process of decoding would compensate for any distortions introduced by display rules.

## Materials and Methods

### Participants

Adult participants were recruited from 4 countries. The countries were selected on an opportunistic basis but Table 1 gives indices and rank positions of the four countries for the relevant Hofstede dimensions. All had been born and raised in their country and were native language speakers. The Northern Ireland (UK) sample consisted of 20 males and 25 females, ranging in age from 19 to 37 years (mean 23.2 years); the Serb sample 25 males and 25 females, ranging in age from 18 to 45 (mean 24.8 years); the Guatemalan sample 39 males and 42 females, ranging in age from 18 to 42 (mean 23.1 years) and the Peruvian sample 30 males and 22 females ranging in age from 18 to 48 (mean 27.5 years). Participants in all samples were a mixture of students and office or manual workers.

**Table 1:** Ranks and raw scores on each of Hofstede's dimensions for the four countries included in the study.

Country	Individualism	Power Distance	Uncertainty Avoidance	Masculinity
Guatemala	4 (6)	1 (95)	1 (101)	4 (37)
Peru	3 (16)	3 (64)	3 (87)	3 (42)
Serbia	2 (25)	2 (86)	2 (92)	2 (43)
N. Ireland	1 (79.5)	4 (31.5)	4 (35)	1 (67)

Northern Irish scores were calculated as a mean score of Great Britain and the Republic of Ireland.

### Ethics Statement

All participants gave written informed consent and the study was approved by the Queen's University Belfast Psychology Research Ethics Committee.

### Stimuli

The 12 stimulus clips were selected from the Belfast Naturalistic Database — an existing database of English language video clips [20]. The clips are originally from a mixture of sources (live interviews, television documentaries and television chat shows) and vary in length from 15 seconds to 75 seconds. All the clips are of conversations between 2 people. Some clips show the posture and body movements of the target individual, some show the reaction of others to the targets behaviour and some provide additional clues regarding context. The clips were selected because the target individuals (5 adult males, 7 adult females) seem subjectively to be displaying a single emotional state (3 sadness, 4 happiness/pleasure/amusement, 4 anger, 1 surprise), although the intensity may vary throughout the clip and there may be blends with other emotions. The clips have English language audio tracks but these were filtered using a notch filter with a lower cut-off of 400Hz and an upper cut-off of between 3,500Hz and 4,000Hz depending on the voice pitch. This was judged to render the speech content unintelligible while retaining most prosodic features.

The stimuli were shown to participants in a pseudo randomised order on a laptop computer using a variant of a computer logging program called FeelTrace [19]. A 10cmx10cm window containing the stimulus appeared on screen alongside an interactive horizontal scale (see Figure 1). Participants used the mouse to move a coloured spot along the scale to trace their changing judgement of the intensity of the emotional expression of the target individual. The bi-directional scale was anchored at the left end by the text “very strongly negative” and at the right by “very strongly positive” with a central “neutral”. Participants were instructed to use the computer mouse to move the dot along the scale to “indicate how strongly you think the person in the video clip is expressing either positive or negative emotion”.

### Design and Procedure

The use of naturalistic stimuli and a computer based trace tool to dynamically rate the valence of expressed emotion is a novel approach to the study of emotion perception. In practice participants found the tool easy to understand and use. All instructions and other communication with participants were in their native language. All participants were tested in the presence of an experimenter. For the Guatemalan and Peruvian participants an interpreter was also present. Following explanation of the task, participants initially used the trace tool while viewing a practice clip as often as they wished. When ready, participants rated the 12 experimental clips. If unhappy with any of their traces, they were free to stop, replay the video clip again and redo the trace as many times as desired until they were satisfied. Only the data from the final attempt was used in the analysis. In practice, this facility was not used very often and seemed mostly restricted to occasions when participants were distracted by something or lost concentration momentarily.

### Data Analysis

The FeelTrace program records the position of the moving spot on the rating scale every 25ms giving a near continuous recording at a rate of 40Hz. An average rating was then calculated for each 0.5 seconds. Scores for the first 3 seconds and for the final 1 second were discarded to eliminate the impact of any variation in the time taken for participants to start recording by holding down the mouse button at the beginning of the clip, or in premature stopping of recording at the end of the clip. This ensured that comparisons were only between active ratings at all times for each participant. The extremes of the scale were scored as +100 and −100 with a score of zero marking the central point. We developed generalized additive mixed models (GAMMs) [21] for each clip. This approach allows non-linear modelling of the trace component of the data while retaining linear fixed effect analysis of effects due to country and sex. No interactions between sex and country were found and were not included in these models. Sex and Country were included as fixed effect factors due to the hypothesised difference in intensities for levels of valence in each country and between sexes. The non-linear smooth terms were also allowed to vary by country using a variable coefficient model [22], [23] due to the importance placed on the hypothesis of universality, although as addressed, this made little contribution to the final models. The traces were not standardized or transformed before fitting the models, the traces for each country within each clip are centred around zero as part of the model fitting process. Participant traces were modelled as random effects with varying intercepts [24]. The error term contains an auto-correlative component and a normally distributed error term. Models were generated using the R package ‘mgcv’ [21].

## Results

All participants rated all 12 video clips. The ratings of the male and female participants in all four countries, averaged over time, show an overall agreed direction of valence for all 12 clips. Five clips are given a mean positive rating and seven a mean negative rating.

### Cross-cultural similarities in valence over the duration of the clips

**Figure 2:**
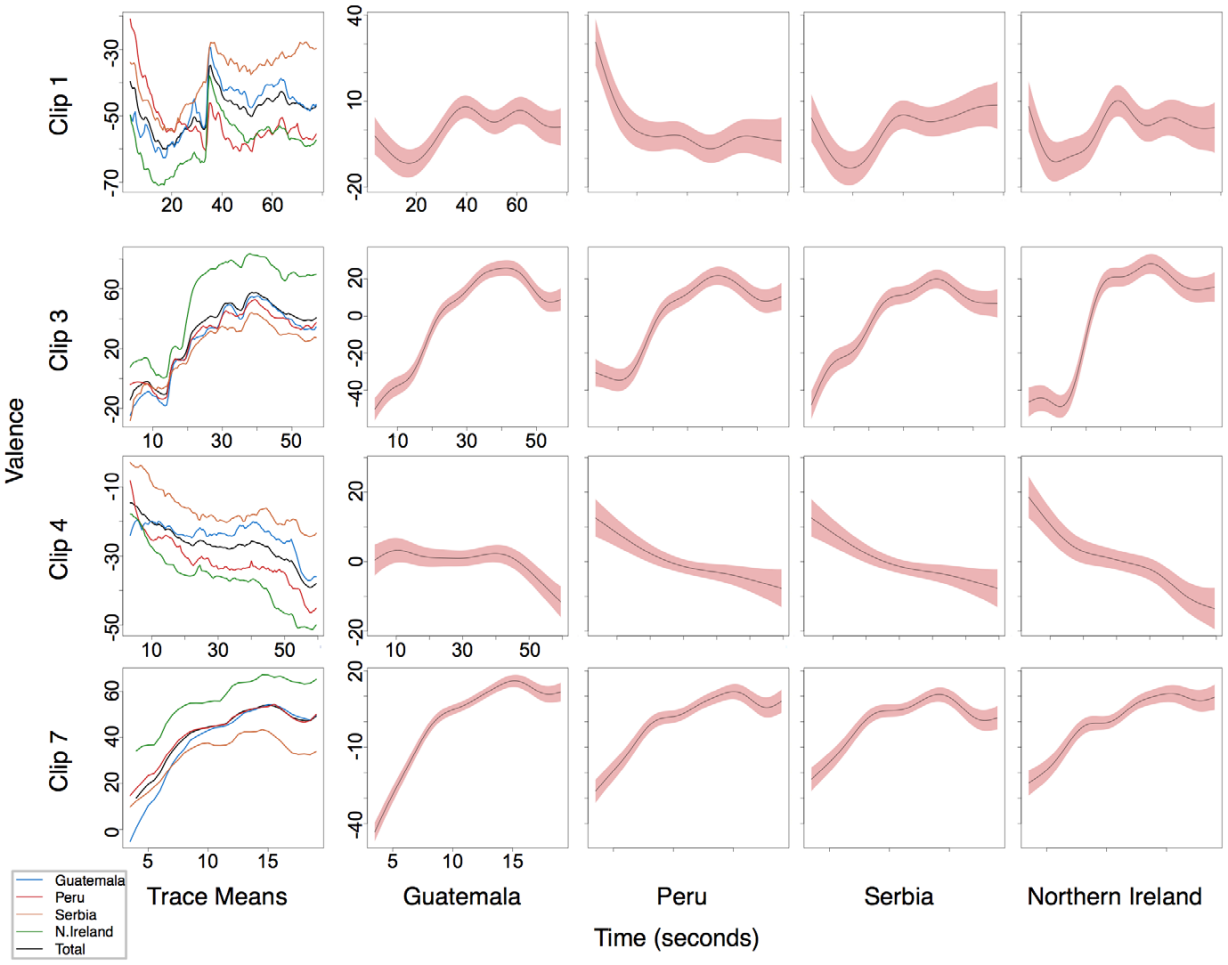
Trace means and generalized additive model terms with coefficients varying for each country for Clips 1, 3, 4 and 7. Shaded red areas represent the 95% confidence intervals. Time in seconds is on the x axis, this differs for each clip. Valence is on the y axis the total range is from −100 to +100 here each clip covers a different range between 50 and 100 units.

Each video clip was analyzed as a separate experiment using generalized additive mixed models [21]. Summary statistics for the clips and corresponding models are presented in Table 2. Four clips — two positive (clip 3 and clip 7) and two negative (clip 1 and clip 4) have been selected for illustration. The means and generalized additive model terms for each country showing change in valence over time can be seen in Figure 2 (Figure S1 and Figure S2 in Supporting Information present the additional clips). Figure 2 clearly displays very similar changes in valence over time for each of the countries. This is confirmed by the high correlations between the countries that can be found in Table 3 (see Table S1 in Supporting Information for additional clip correlations). The generalized additive model terms, although very similar, include a correction for autocorrelation in the time series. Table 2 shows that there are no differences in intensity of rating for sex in any of the clips.

**Table 2:** Summary statistics for the twelve clips and associated statistical models.

Clip	time(secs)	Valence	Target sex	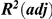	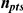	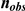	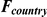	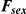
1	75	−ve	female	0.08	228	33972	6.26***	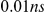
2	15	+ve	male	0.16	226	4746	6.71***	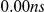
3	64	+ve	female	0.42	224	24192	25.28***	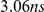
4	60	−ve	female	0.11	228	25764	8.15***	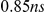
5	16	+ve	female	0.22	227	5675	13.34***	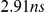
6	15	+ve	male	0.11	226	4972	7.80***	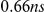
7	21	+ve	female	0.22	227	7264	6.58***	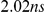
8	30	−ve	male	0.03	226	11300	0.97*ns*	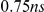
9	33	−ve	male	0.08	227	13393	7.64***	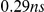
10	18	−ve	Female	0.10	227	6583	2.92*	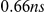
11	30	−ve	female	0.09	224	11872	7.66***	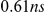
12	21	−ve	male	0.10	220	7040	3.11*	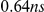

Target sex is the sex of the person at the focus of attention in the video clip, 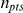 is the number of participants, differences are due to incomplete sessions. 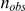 is the total number of observations used in the model. Significance codes:

*p<0.05,

**p<0.01,

***p<0.001.

**Table 3:** Correlation matrices for Clips 1, 3, 4 and 7.

	Guatemala	Peru	Serbia	N. Ireland
Clip 1				
Guatemala	—	−0.48***	0.88***	0.94***
Peru	−0.24**	—	−0.28***	−0.23**
Serbia	0.85***	−0.15	—	0.85***
N. Ireland	0.93***	−0.06	0.83***	—
Clip 3				
Guatemala	—	0.99***	0.99***	0.98***
Peru	0.99***	—	0.97***	0.99***
Serbia	0.99***	0.97***	—	0.97***
N. Ireland	0.97***	0.98***	0.97***	—
Clip 4				
Guatemala	—	0.68***	0.62***	0.74***
Peru	0.74***	—	0.96***	0.96***
Serbia	0.63***	0.92***	—	0.97***
N. Ireland	0.76***	0.94***	0.90***	—
Clip 7				
Guatemala	—	1.00***	0.96***	0.99***
Peru	1.00***	—	0.97***	0.99***
Serbia	0.96***	0.97***	—	0.93***
N. Ireland	0.98***	0.99***	0.91***	—

Correlations for fitted values of the generalized additive model terms are in the upper triangle while those for the means traces are in the lower triangle. Significance codes:

*p<0.05,

**p<0.01,

***p<0.001.

### Differences between countries in intensities of positive clips

There are significant main effects of country in all but one of the models (clip 8). This main effect can be loosely conceptualised as an overall difference in the intensity of valence scores. This effect is most obvious in Figure 2 where the positive trace means for clips 3 and 7 show a marked increase in valence for Northern Ireland but retain the overall shape of the curve. These effects are displayed in the main effects statistics for country in Table 2.

Multiple comparisons indicate some patterns in the differences between countries. Multiple comparisons use paired t-test with p-values adjusted for familywise error using the Holm procedure [25]. Results are reported for each clip in Table 4. The clearest pattern occurs in the five positive valence clips. For positive clips the most consistent pattern places Northern Ireland as the most positive, Serbia as the least positive and Guatemala and Peru fall in between them and only differ in two of the clips. Furthermore the explanatory value of the positive models is much greater than the negative models. All five of the positive models explain more than 11% of the variance in the data (16%, 42%, 22%, 11%, 22%) while none of the negative models succeeds in explaining more than 10% of the variance.

**Table 4:** Multiple Comparisons between countries.

Positive Clips	Most Positive			Least Positive
2	N. Ireland	Peru 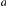	Guatemala 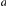	Serbia
3	N. Ireland	Peru 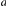	Guatemala 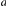	Serbia
5	N. Ireland	Guatemala 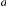	Serbia 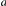	Peru 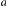
6	N. Ireland	Guatemala	Peru 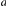	Serbia 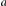
7	N. Ireland	Peru	Guatemala	Serbia
Negative Clips	Most Negative			Least Negative
1	N. Ireland	Peru	Guatemala	Serbia
4	N. Ireland	Peru	Guatemala	Serbia
8	Peru	Serbia	N. Ireland 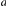	Guatemala 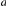
9	Guatemala	Peru	Serbia 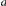	N. Ireland 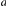
10	N. Ireland 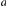	Guatemala 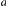	Peru	Serbia
11	Peru	Guatemala	N. Ireland	Serbia
12	N. Ireland 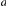	Guatemala 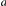	Peru 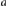	Serbia

Countries within a row sharing a common subscript are not significantly different at 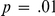 (Holm, 1979).

### Differences between countries in the intensities of negative clips

The pattern is less clear in the negative clips (Table 4). The most prominent pattern places Northern Ireland as the most extreme but this time with the most extreme negative scores and again Serbia is most often the closest to zero. In four of the seven negative clips Northern Ireland produces the most negative ratings and Serbia the least negative ratings. The patterns in clips 1 and 4 reflect the patterns for the positive clips and to some extent the same can be said of the response patterns for clips 10 and 12, with less consistency for Peru and Guatemala. The response patterns for clips 9, 11 and 8 are somewhat more ambiguous.

## Discussion

The remarkable agreement shown by male and female participants from four different countries in their overall rating patterns while viewing natural emotional behaviour offers strong support for the universality of emotion judgements. To the best of our knowledge the present study provides the first evidence that the previously reported interplay between universal and cultural influences extends to ratings of natural, dynamic emotional stimuli. The statistical model used to analyse the data varied across clips in the extent to which it could account for the sources of the variance in the rating patterns. This is not surprising. Although the rating task may seem straightforward, raters are actually faced with a complex series of decisions as they attempt to transform what they see on the screen into a continuous stream of scores recorded by the computer. It is likely that individuals will vary in their appraisal of what is happening in the scene, in their judgement of the emotional behaviour of the target individual, in their understanding of the terms ‘positive emotion’ and ‘negative emotion’ and in their movement of the computer mouse to translate their rating into a point on the onscreen scale. Recent evidence indicates that short term physiological changes [26] and longer term psychological characteristics [27] can also influence the nature of our perception of faces. The clips undoubtedly vary in the clarity of the contextual information offered to raters, as do real life emotional situations. Some of the interactions depicted in the films seem easy to interpret quickly, allowing greater consensus between raters, while others may remain rather ambiguous.

Nevertheless, systematic differences did emerge in the intensity levels at which participants from the different countries rated the behaviours. In all five of the films where behaviour was rated as having positive valence, the Northern Irish sample rated the emotion as at a higher intensity than raters from any other country. This offers support for the suggestion [16] of an in-group advantage. Although the clips vary in their nature, the target individuals performing the emotional behaviour are all based in the UK. Our results indicate that the temporal pattern of ratings appears similar across cultures but that mean intensity ratings show systematic variation. Perhaps the most straightforward explanation for this combination of findings is that decoders from different cultures understand the gist of what is happening, but they fail to grasp the more subtle nuances. This suggestion has been further developed [12] to include the idea that subtle variations in ‘dialects’ of emotional communication result in in-group advantage, and that with increasing cultural and geographical distance between two countries, the more scope there is for misunderstanding. Our results offer only limited support for this.

In the present study, the presence of a filtered vocal soundtrack accompanying the visual stimulus may have reduced some of the differences between the cultural groups. A meta-analysis of studies of emotion recognition [7] indicated that adding sound to a silent channel reduced the in-group advantage. It has been suggested [28] that listeners can rapidly and accurately identify emotion from vocal tone, and that similar brain regions may be implicated in processing the emotional cues in the face and in the prosodic features of the voice. It has also been reported [29] that although it is often possible to judge emotion from the face or from the voice, both speed and accuracy of judgement increase when both are expressing the same emotion — an effect that persists even when participants are instructed to ignore the vocal channel [30], suggesting that extracting emotional information from the voice may be automatic. It is possible therefore that the addition of (in this case degraded) vocal information may make the task of judging emotion easier for all groups. However, a cross-cultural study of vocal emotion judgements [31] found that when meaningless sentences were read in different emotional styles by German actors, participants whose language was more distant from German were less accurate. They suggest that much emotional information may be carried in vocal features such as rhythm, timing and vocal inflection which may give an advantage to those judges whose language shares those features. Although the cultures in the present study are geographically disparate, their languages all share an Indo-European root.

Although Guatemalan and Peruvian samples showed differences from each other in rating 8 of the 12 clips, and significantly differed from the Northern Irish sample on 10 (Guatemala) or 11 (Peru) of the clips, it was the Serbian sample who showed the greatest difference in ratings when compared to the Northern Irish (all 12 clips). We would have expected the ratings of the two Latin American cultures to differ more systematically from those of the two European cultures based on their cultural and geographical distances.

Another possibility is that people from the four countries are judging the clips differently because they are applying knowledge of their own display rules to the ratings [13]. Indeed, one stimulus for this research was the observation by one of the authors (TV) that people from Northern Ireland seemed much less emotionally demonstrative than people from Serbia. If it is true that the normative level of emotional expression is lower in Northern Ireland than in Serbia (perhaps because high levels of emotional expression are discouraged), then one might intuitively expect observers from Northern Ireland to judge any given case of emotional expression against this lower norm and thus give it a higher rating. Our results for all five of the positively valenced clips and for four of the seven negatively valenced clips support this suggestion — the Northern Irish sample rated both positive and negative emotion at a significantly more extreme level than the Serb sample. Although this result may support the possibility that there are different sets of display and decoding rules operating in the two countries, it seems to support the idea that this particular decoding rule operates by correcting any distortion introduced by the display rule, rather than by introducing further distortion. However there is less support for the detailed hypotheses [6], [10] relating Hofstede's cultural dimensions to emotion recognition. The four countries in this study were allocated scores on the two potentially relevant dimensions (Individualism and Power Distance) of Hofstede's system [9] — the scores for Northern Ireland, a culturally divided region of the United Kingdom were averaged from those of the U.K. and Ireland, which are already similar. Northern Ireland has a much higher Individualism index and a much lower Power Distance index than the other countries. Both factors should influence their ratings of negative emotions more than positive emotions. In fact the most consistent differences between the Northern Irish ratings and those of the other countries were on the positively valenced clips. The negatively valenced clips showed more varied results.

This first study using these methods was intended to reveal how participants from different cultures would rate a range of video clips that varied in their length, in the type and strength of emotion displayed and in the sex of the displaying individual. The differences that have emerged in the response patterns shown to these twelve short slices of emotional life — the finding that the clips differ in the levels and, in some cases, the patterns of cross cultural agreement they elicit — indicate additional intriguing research questions. The finding that for nine of the twelve clips the Northern Ireland participants rated the emotion as more strongly negative or more strongly positive than their Serbian counterparts, raises the question ‘what distinguishes these clips from the remaining three clips?’ Unfortunately answering this question is beyond the scope of the present study — there are too few clips of each type to allow a definitive answer.

The use of valence as the rating measure can only be a first step in using dynamic spontaneous stimuli to investigate cultural differences in the perception of the facial display of emotion. We have begun with valence ratings to ascertain whether universality of valence judgements could be established using such novel methods before moving on to ratings of intensities of discrete emotions. In a critique of the universal recognition hypothesis Russell [17] suggested that agreement on dimensions such as valence and arousal may be universal, but that recognition of more discrete categories of emotion such as happiness or anger may be culture specific. Results from the present study strongly support the view that there is a high degree of cross cultural agreement on ratings of valence. However, it is important to follow this study with similar studies focused on discrete emotions. We believe that the present methods can be adapted to gather such evidence and it is not at all clear if similar temporal patterns of cross-cultural agreement will emerge when we ask participants to continuously rate the level of a discrete emotion such as anger or fear.

The degree to which the outward behaviour of each target individual in the clips accurately reflects their inner emotional state is highly likely to vary across the clips and within each clip over time, for a variety of reasons. Each clip is a recording of a social interaction between the target individual and at least one other person, usually in the presence of others. We should therefore expect to see expression of emotion interfered with by a range of facial and bodily behaviours that are part of normal communication and need not be conveying any information about emotion [8]. At any moment individuals may, consciously or unconsciously, also be modifying (attenuating, masking, substituting or exaggerating) their expression of emotion for a variety of reasons. These are all features of real life social interaction in which we, as participants or observers, try to make judgements about the emotional state of another person. This study can be criticised because the video clips we have used do not contain experimentally controlled performances in which we can be objectively certain about what is going on. However, the very fact that the stimuli are varied and uncontrolled slices of emotional life makes the agreement between men and women across four quite different countries all the more remarkable. It appears that the use of such uncontrolled stimulus material may be capable of yielding unsuspected insights into the similarities and differences in the way people from different cultures make sense of the emotional lives of others.

## Supporting Information

Table S1 Correlation matrices for fitted values of the generalized additive model terms and mean traces in clips 2, 5, 6, 8, 9, 10, 11 and 12. Correlations for generalized additive model terms are in the upper triangle while those for the means are in the lower triangle.
(0.05 MB PDF)

Figure S1 Trace means and generalized additive model terms with coefficients varying for each country for Clips 2, 5, 6 and 8. Shaded red areas represent the 95% confidence intervals. Time in seconds is on the x axis, this differs for each clip. Valence is on the y axis the total range is from −100 to +100 here each clip covers a different range between 50 and 100 units.
(0.54 MB TIF)

Figure S2 Trace means and generalized additive model terms with coefficients varying for each country for Clips 9, 10, 11 and 12. Shaded red areas represent the 95% confidence intervals. Time in seconds is on the x axis, this differs for each clip. Valence is on the y axis the total range is from −100 to +100 here each clip covers a different range between 50 and 100 units.
(0.56 MB TIF)
