# Supplementary material for: Cross-Cultural Patterns in Dynamic Ratings of Positive and Negative Natural Emotional Behaviour
Source: PLoS One. 2011 Feb 18;6(2):e14679. doi: 10.1371/journal.pone.0014679 (PMC3041750; doi:10.1371/journal.pone.0014679)
Supplement: Table S1 — Correlation matrices for fitted values of the generalized additive model terms and mean traces in clips 2, 5, 6, 8, 9, 10, 11 and 12. Correlations for generalized additive model terms are in the upper triangle while those for the means are in the lower triangle. (0.05 MB PDF) [file pone.0014679.s001.pdf]

Table S1. Correlation matrices for fitted values of the generalized additive model terms and mean traces in clips 2, 5, 6, 8, 9, 10, 11 and 12. Correlations for generalized additive model terms are in the upper triangle while those for the means are in the lower triangle

	Guatemala	Peru	Serbia	N. Ireland
Clip 2				
Guatemala	—	0.93***	0.99***	0.99***
Peru	0.91***	—	0.90***	0.94***
Serbia	0.98***	0.91***	—	0.98***
N. Ireland	0.99***	0.94***	0.98***	—
Clip 5				
Guatemala	—	0.95***	0.99***	0.96***
Peru	0.94***	—	0.91***	0.98***
Serbia	0.99***	0.91***	—	0.92***
N. Ireland	0.93***	0.97***	0.89***	—
Clip 6				
Guatemala	—	0.24	0.73***	0.41**
Peru	0.27	—	0.80***	0.93***
Serbia	0.60**	0.80***	—	0.92***
N. Ireland	0.48*	0.88***	0.96***	—
Clip 8				
Guatemala	—	−0.68***	−0.84***	−0.68***
Peru	−0.54***	—	0.92***	0.97***
Serbia	−0.67***	0.87***	—	0.90***
N. Ireland	−0.58***	0.91***	0.81***	—
Clip 9				
Guatemala	—	−0.54***	0.78***	0.96***
Peru	−0.44***	—	0.00	−0.52***
Serbia	0.74***	0.09	—	0.84***
N. Ireland	0.90***	−0.40**	0.84***	—
Clip 10				
Guatemala	—	0.96***	0.95***	0.96***
Peru	0.96***	—	0.99***	1.00***
Serbia	0.93***	0.98***	—	0.99***
N. Ireland	0.95***	0.98***	0.98***	—
Clip 11				
Guatemala	—	−0.12	0.43**	0.34*
Peru	−0.05	—	−0.19	0.84***
Serbia	0.23	−0.09	—	0.23
N. Ireland	0.44**	0.71***	0.40**	—
Clip 12				
Guatemala	—	0.77***	0.93***	0.88***
Peru	0.87***	—	0.65***	0.98***
Serbia	0.91***	0.71***	—	0.77***
N. Ireland	0.93***	0.98***	0.80***	—

Correlations for fitted values of the generalized additive model terms are in the upper triangle while those for the means traces are in the lower triangle. Significance codes: '*' $p < .05$, '**' $p < .01$, '***' $p < .001$
