# Supplementary figures and images for: Cross-Cultural Patterns in Dynamic Ratings of Positive and Negative Natural Emotional Behaviour
Source: PLoS One. 2011 Feb 18;6(2):e14679. doi: 10.1371/journal.pone.0014679 (PMC3041750; doi:10.1371/journal.pone.0014679)

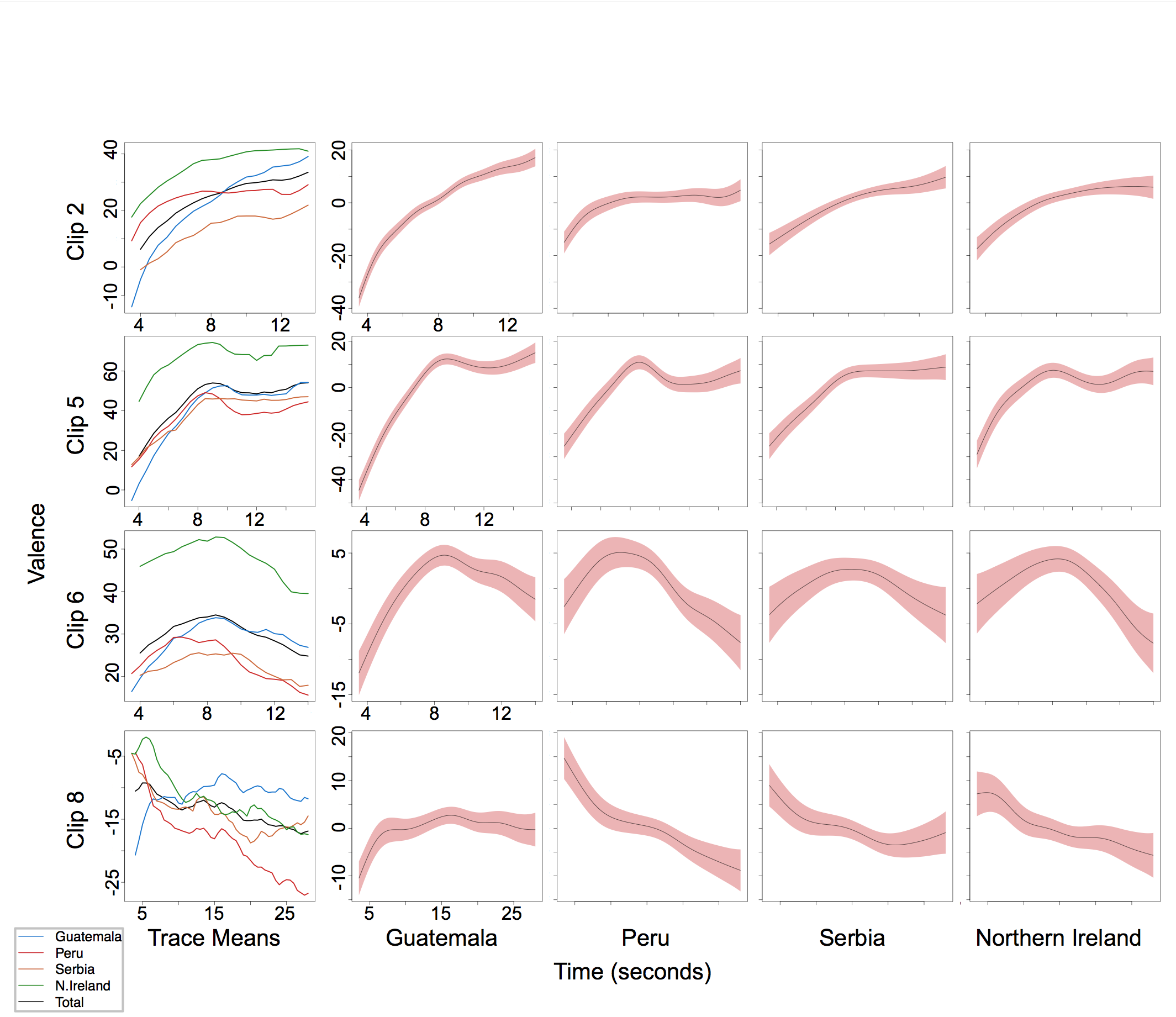

Supplement: Figure S1 — Trace means and generalized additive model terms with coefficients varying for each country for Clips 2, 5, 6 and 8. Shaded red areas represent the 95% confidence intervals. Time in seconds is on the x axis, this differs for each clip. Valence is on the y axis the total range is from −100 to +100 here each clip covers a different range between 50 and 100 units. (0.54 MB TIF) [file pone.0014679.s002.tif]

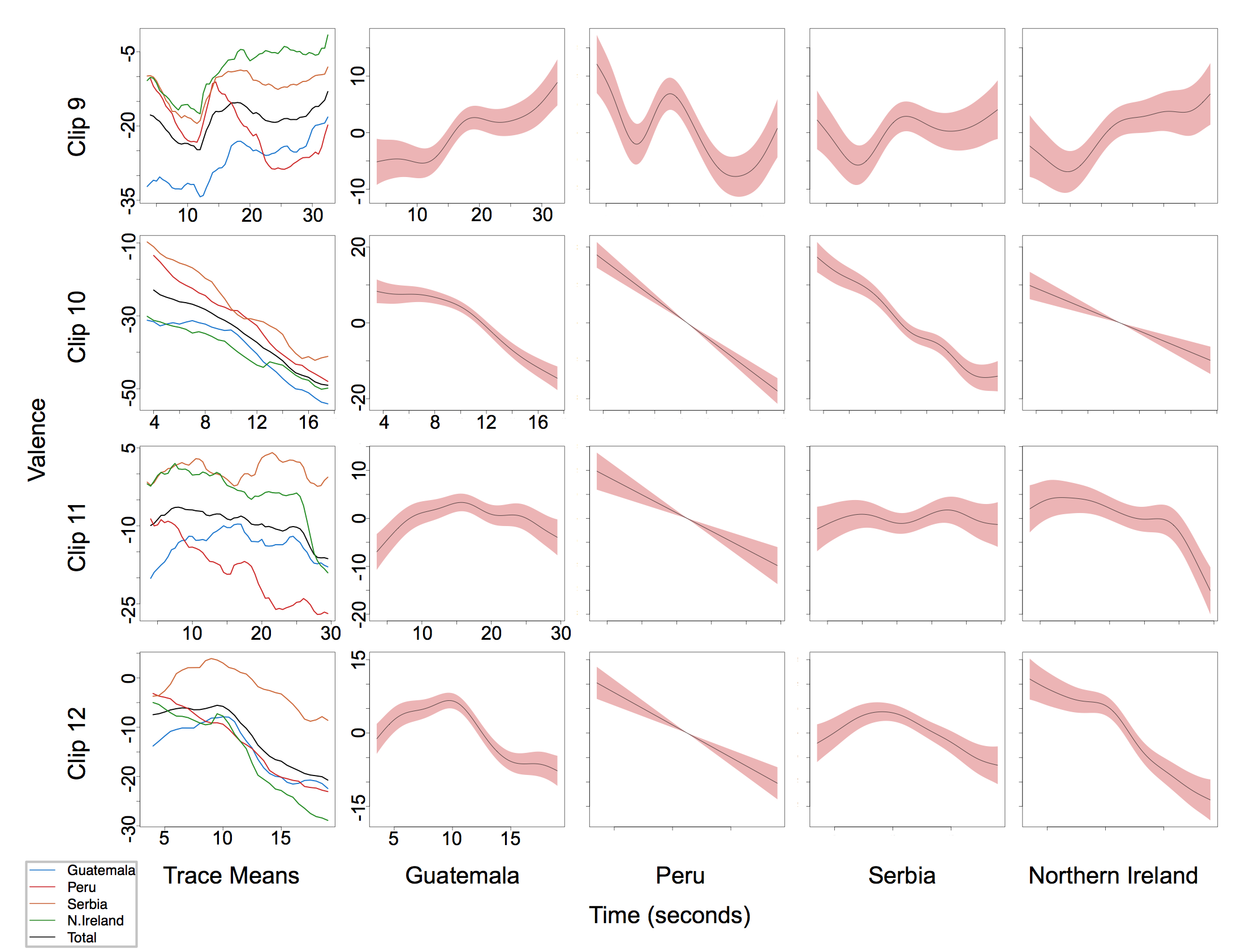

Supplement: Figure S2 — Trace means and generalized additive model terms with coefficients varying for each country for Clips 9, 10, 11 and 12. Shaded red areas represent the 95% confidence intervals. Time in seconds is on the x axis, this differs for each clip. Valence is on the y axis the total range is from −100 to +100 here each clip covers a different range between 50 and 100 units. (0.56 MB TIF) [file pone.0014679.s003.tif]
